# Two-institution results of Stereotactic Body Radiation Therapy (SBRT) for treating adrenal gland metastases from liver cancer

**DOI:** 10.1186/s12885-023-10519-9

**Published:** 2023-01-21

**Authors:** Bichun Xu, Xianzhi Zhao, Di Chen, Wenjuan Zhao, Xiaoyan Wang, Changhua Ding, Zhiyong Yuan, Huojun Zhang

**Affiliations:** 1 Department of Radiation Oncology, Shanghai Changhai Hospital, the Navy Medical University, 200433 Shanghai, China; 2 Department of Radiation Oncology, Key Laboratory of Cancer Prevention and Therapy, Tianjin’s Clinical Research Center for Cancer, Tianjin Medical University Cancer Institute & Hospital, National Clinical Research Center for Cancer, 300060 Tianjin, China

**Keywords:** Adrenal gland metastases (AGM), Stereotactic body radiotherapy (SBRT), Liver cancer, Local control, Survival

## Abstract

**Objective:**

Stereotactic Body Radiation Therapy (SBRT) has been found beneficial for adrenal gland metastases (AGMs) with a high local control rate and low toxicity. The role of SBRT for AGMs in patients with liver cancer has not been well-discussed before. We, therefore, report our two-institution experience to further elaborate on the feasibility and effectiveness of SBRT in the treatment of AGMs from liver cancer.

**Methods:**

A total of 23 liver cancer patients (19 males, 4 females) with 24 AGMs treated by SBRT from July 2006 to April 2021 were retrospectively included in this study. Toxicity was assessed based on clinical adverse events using the Common Terminology Criteria for Adverse Events (CTCAE) version 5.0. The effectiveness was assessed based on local control (LC), progression-free survival (PFS), and overall survival (OS), which were calculated using the Kaplan-Meier method. Univariate analyses were compared by log-rank test. The relevant covariates were evaluated using Cox proportional hazards models.

**Results:**

The median dose was 40 Gy in 5 fractions, with the corresponding median biological effective dose (BED10, α/β = 10 Gy) of 72 Gy. The median overall follow-up time was 15.4 months (range: 4.2–70.6 months). The complete response (CR), partial response (PR), stable disease (SD) and progressive disease (PD) rates were 25.0%, 20.8%, 33.3%, and 20.8%, respectively. All 6 patients with AGMs accompanying symptoms had varying degrees of alleviation after SBRT. The 0.5-, 1-year and 2-year LC rates were 87.5%, 77.8%, and 77.8%, respectively. The 0.5-, 1-year and 2-year OS rates were 95.5%, 66.8%, and 41.1%, respectively. The treatments were all tolerated with only one patient reporting a grade-3 hepatic injury. The univariate analysis concluded that only gross tumor volume (GTV) < 34.5 ml (*p* = 0.039) was associated with a favorable LC rate. After multivariate analysis, favorable predictors correlated with OS were GTV < 34.5 ml (*p* = 0.043), systemic therapy (*p* = 0.017), and without additional organ metastasis after SBRT (*p* = 0.009).

**Conclusion:**

Our results suggest that SBRT is a safe and effective technique to treat AGM from liver cancer, especially for small GTV (< 34.5ml). Moreover, the small metastatic lesion volume, fewer metastatic lesions, and intervention of systemic therapy are more likely to improve OS.

## Background

According to a recent statistics report in China, liver cancer remains one of the most common causes of cancer mortality due to its high aggressiveness and prevalence in East Asian populations [1]. Liver cancer cells can spread throughout the body directly or indirectly through lymphatic and vascular pathways, resulting in simultaneous or heterochronic extrahepatic metastases (EHM). The development and deterioration of EHM have predicted a poor prognosis for liver cancer patients [2]. Generally, AGM is the third most common EHM from liver cancer (followed by lung and bone), accounting for 6.9–19.1% of patients with EHM [3]. Some patients with AGM may feel a sense of swelling distension, soreness in the kidney area, and back pain, which lead to a reduced quality of life (QoL) and even affect their survival.

Numerous therapies have been applied to treat AGM, but there is still no universally recognized treatment. With the advancement of systemic therapies such as immunotherapy and targeted therapy in recent years, more liver cancer patients with distant metastatic lesions may have a better chance of an improved OS. Currently, the best therapeutic option for progressive hepatocellular carcinoma is a combination of programmed death-ligand 1(PDL1) blockers and vascular endothelial growth factor(VEGF) antagonists, whereas chemotherapy remains the best alternative for intrahepatic cholangiocarcinoma [4]. However, resistance to the drugs and heterogeneity in patient response limit their widespread use. Moreover, they have a limited effect on the local control rate of lesions compared with local therapy [5]. Therefore, more aggressive local treatment options are proposed to reduce the growing tumor burden. Surgery is considered a curative option for isolated AGM, whereas contraindications such as the senior, poor cardiopulmonary function, and other co-morbidities may prevent it from conducting. Moreover, adrenalectomy is an invasive treatment for patients [6]. Other loco-regional approaches, including transcatheter arterial chemoembolization (TACE), percutaneous ethanol injection (PEI), and radiofrequency ablation (RFA) are not suitable for large lesions, which may cause stenosis, fistulas, and bleed [7]. Thus, there is an urgent need for a precise, reliable, and effective option.

The appropriate management of radiotherapy iseffective in relieving pain symptoms as well as improving survival in patients with AGMs from liver cancer [8–11]. Radiotherapy techniques have been evolving, while stereotactic body radiation therapy (SBRT) can achieve better conformality, higher accuracy, and better dose distribution compared to conventional radiotherapy [12]. SBRT could be well delivered with the advanced devices represented by the CyberKnife® system (Accuray Inc., Sunnyvale, CA, USA). The SBRT technique has been acknowledged as a non-invasive but curable treatment alternative. Distinguishing features of the CyberKnife® system include near real-time tracking via position information, the flexibility of robotic beam movement, and delivery capability [13–15].

There are few reports on SBRT applied in AGM from liver cancer, which mainly have adopted conventional radiotherapy [8–11]. In addition, previous studies of AGMs SBRT have focused on various solid tumors as primary tumors, and no report on the efficacy of SBRT in the treatment of AGMs from liver cancer. Therefore, the purpose of this retrospective two-center study is to evaluate the efficacy and safety of SBRT for AGMs from liver cancer.

## Methods

### Patient characteristics

From July 2006 to April 2021, SBRT was administered to 23 patients with 24 AGMs from liver cancer at Tianjin Medical University Cancer Institute and Hospital, and Shanghai Changhai Hospital of the Navy Medical University. Prior to the enrollment and treatment, all patients signed an informed consent form and received thorough information from their oncologists regarding the potential toxicity and benefits of SBRT. The inclusion criteria for this retrospective study were as follows: (1) The diagnosis of liver cancer [16]; (2) AGMs diagnosed by pathological biopsy or clinically by at least one imaging modality, including positron emission tomography /computed tomography (PET/CT), enhanced computed tomography (CT), enhanced magnetic resonance imaging (MRI); (3) Karnofsky performance score ≥ 70; (4) Completion of planned SBRT; (5) A life expectancy of more than 3 months; (6) Patients who rejected, could not tolerate, or were unfit for surgery or other invasive treatments were also included in the study.

In addition, written informed consents had been obtained from all patients prior to the treatment, stating their willingness to be treated according to the protocol. This retrospective study was reviewed and approved by the Ethics Committees of both Shanghai Changhai Hospital of the Navy Medical University and Tianjin Medical University Cancer Institute and Hospital, it was conducted according to the Declaration of Helsinki.

### Delivery of SBRT

All SBRT treatments were performed using the 6MV highly accurate CyberKnife (Accuray Corporation Sunnyvale, CA, USA) linear accelerator. Patients were immobilized in a supine position with arms by their sides using vacuum bags. Enhanced computed tomography (CT) scan was performed with a slice thickness of 1.5 mm at least 10 cm above and below the tumor. The gross tumor volume (GTV) was defined as a radiographic lesion based on contrast-enhanced CT, FDG PET/CT, or MR scans. The average respiratory motion of adrenal gland metastases in left-right (LR), cranial-caudal (CC), anterior-posterior (AP) directions was 3.4 ± 2.2 mm, 9.5 ± 5.5 mm, and 3.8 ± 2.0 mm, respectively [17]. According to the metastases motion, planning target volume (PTV) was delineated with a 3–5 mm margin expansion in LR and in AP direction respectively, and a 3–8 mm margin expansion in CC direction from GTV. The Synchrony™ Respiratory Tracking System was used for 2 lesions in 2 patients, while the X-sight Spine Tracking System was used for 22 lesions in 21 patients. Seven of the 23 patients (7 lesions) were treated for radical purposes while 16 patients (17 lesions) were for palliative purposes, mainly for pain relief and tumor burden reduction. Treatment protocols were determined by the oncologists depending on patients’ heterogeneity. The SBRT was generally administered at the maximum safe dose, as a higher biologically effective dose (BED) may be associated with an improved local control rate. The dose-volume constraints for organs at risk were referred to the American Association of Physicists in Medicine guidelines in TG-101 [18].

### Response evaluation and follow-up

Local tumor response was assessed according to Response Evaluation Criteria in Solid Tumors (RECIST) version 1.1, including CR, PR, SD, and PD [19]. LC was defined as CR, PR, and SD. LC was the length of time from the date of initiation of SBRT to the date of local tumor progression or last follow-up. OS was estimated from the date of initiation of SBRT to the date of death or last follow-up. PFS was the time interval between the start of SBRT and the date of the detection of progression at any site or death by any cause or the last follow-up. Each patient was assessed by an oncologist for side effects such as nausea, abdominal pain, or poor appetite, and was graded according to the Common Terminology Criteria for Adverse Events (CTCAE) v5.0. Acute toxicity was defined as side effects occurring between the start of SBRT to three months after SBRT, while those occurrences three months after SBRT were defined as chronic toxicity.

### Statistical analysis

LC, OS, and PFS curves were plotted using the Kaplan-Meier method. Potential factors associated with LC rate and OS rate were identified with univariate log-rank comparisons, then multivariate Cox proportional hazards regression model. Factors with *p*-values < 0.1 in the univariate analysis were included in the multivariate analysis. The statistical analysis was performed with SPSS version 26.0 and a two-sided *P* value < 0.05 was considered statistically significant.

## Results

### Patient characteristics

A total of 23 patients with 24 lesions were included in this study. The median age of the study cohort was 54 years (range: 42–74 years old). The median time between the initial diagnosis of liver cancer and the diagnosis of adrenal metastasis was 23.2 months (range: 1.3–86.9 months). Eighteen of these lesions were located in the right adrenal gland and four in the left adrenal gland, while one patient had bilateral lesions. Pathological types of primary tumor included hepatocellular carcinoma (*n* = 17, 73.9%), intrahepatic cholangiocarcinoma (*n* = 4, 17.4%), and others not obtained (*n* = 2, 8.7%). Among the fourteen patients with concomitant metastases at other sites, 10 (71.4%) patients received SBRT for both adrenal gland and other sites’ lesions. Systemic therapy, such as targeted therapy or immunotherapy treatment, was given to eight of all patients (34.8%). Detailed information on patient characteristics was shown in Table 1. In addition, 17 patients (73.9%) were asymptomatic, while 6 (26.1%) had abdominal pain, back pain, and flank pain. The median GTV was 33.09 ml (range: 3.38 ml-180.5 ml), the median prescribed dose was 40 Gy (range: 33–52 Gy) in 5 fractions (3–8 fractions) and the corresponding biologically effective dose (BED) (α/β = 10 Gy, BED_10_) was 72 Gy (range: 53.7-100.8 Gy). The treatment parameters were displayed in Table 2.

**Table 1:** Patients demography and clinical presentation

Characteristics	Values
Age (years)	Median 54 (range 42-74)
Gender (male/female)	19/4 (82.6%/17.4%)
Karnofsky performance score ≥ 70	23 (100%)
Pathology of primary tumor	
▪ Hepatocellular carcinoma	17 (73.9%)
▪ Intrahepatic cholangiocarcinoma	4 (17.4%)
▪ N/O	2 (8.7%)
Lesions per patient	
▪ 1	22 (95.7%)
▪ >1	1 (4.3%)
Symptoms	
▪ Presented	6 (26.1%)
▪ None	17 (73.9%)
Location of AGMs	
▪ Left	4 (17.4%)
▪ Right	18 (78.3%)
▪ Left and right	1 (4.3%)
Systemic therapy	
▪ Yes	8 (34.8%)
▪ None	15(65.2%)
Metastases in other sites (at the same time)	
▪ Yes	14 (60.9%)
▪ None	9 (39.1%)
SBRT for other sites (at the same time)	
▪ Yes	10 (43.5%)
▪ None	13 (56.5%)

*N/O* not obtained, *AGMs* adrenal gland metastases, *SBRT* stereotactic body radiation therapy

**Table 2:** Treatment parameters used for SBRT

	All lesions	Lesions with local control	Lesions without local control
Maximum dose (Gy)	57.4 (44-73.2)	57.7 (44-73.2)	57.1 (51.9-64.3)
Total prescribed dose (Gy)	40 (33-52)	41.6 (33-52)	40 (40-45)
Number of fractions	5 (3-8)	5 (3-8)	5 (5-8)
Dose per fraction (Gy)	8 (4.6-14)	7.8 ( 4.6-14)	8 (5-9)
BED_10_ (Gy)	72 (53.7-100.8)	72 (53.7-100.8)	72 (60-85)
Prescription isodose line (%)	73 (66- 78)	73 (68-78)	70 (66-77)

All data were shown as median values (range). BED_10_ : biologic equivalent dose (α/β=10 Gy)

### Efficacy outcomes

**Fig. 1:**
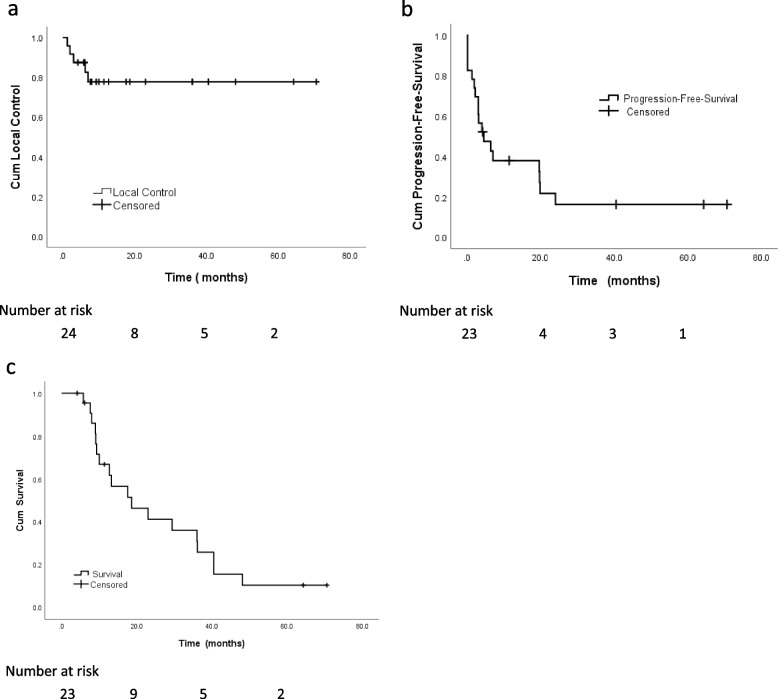
Actuarial survival analysis of patients. **a** Overall local control. **b **Overall progression free survival. **c **Overall survival

The median follow-up was 15.4 months (range: 4.2–70.6 months). Based on the RECIST criteria, the CR, PR, SD, and PD rates were 23.8%, 23.8%, 31.0%, and 21.4%, respectively. The 0.5-, 1-year and 2-year LC rates were 87.5%, 77.8%, and 77.8%, respectively (Fig. 1a). A total of 4 patients with 5 targeted lesions showed local progression, 3 lesions progressed within 6 months after SBRT and the remaining two lesions were 7 months and 7.6 months, respectively. The mean LC was 55.8 months (95%CI 44.3–67.3 months). All 6 patients with AGMs accompanying symptoms had varying degrees of alleviation after SBRT. In the univariate analysis, for 13 targets with GTV < 34.5 ml, the 2-year LC rates were 92.3% while for 11 targets with GTV ≥ 34.5 ml, the 2-year LC rates were 58.2% (*P* = 0.039). Notably, simultaneous irradiation of other metastatic lesions may increase the rate of local control, but the correlation was not remarkable (*p* = 0.056). However, no other predictors were associated with LC after univariate analysis (Table 3). Furthermore, no prognostic factors were found to be correlated with the LC rate after the multivariate analysis.

**Table 3:** Univariate analysis for LC rate

	0.5-year LC rate (%)	1-year LC rate (%)	2-year LC rate (%)	*P* value
BED_10_ (Gy)				
▪ < 75	85.7	66.7	66.7	0.260
▪ ≥ 75	90.0	90.0	90.0	
Prescription dose (Gy)				
▪ < 41.6	84.6	63.5	63.5	0.179
▪ ≥ 41.6	90.9	90.9	90.9	
GTV (ml)				
▪ < 34.5	100	92.3	92.3	0.039
▪ ≥ 34.5	72.7	58.2	58.2	
Gender				
▪ Male	85.0	72.9	72.9	0.268
▪ Female	100	100	100	
Tracking method				
▪ Synchrony respiratory motion	100	100	100	0.461
▪ X-sight spine	84.6	75.6	75.6	
Location of AGMs				
▪ Left	100	80	80	0.860
▪ Right	84.2	77.7	77.7	
Systemic therapy				
▪ Yes	87.5	87.5	87.5	0.492
▪ None	87.5	72.9	72.9	
SBRT for other sites				
▪ Yes	100	100	100	0.056
▪ None	78.6	64.3	64.3	

*LC* local control, *BED*_*10*_ biologic equivalent dose (α/β=10 Gy), *GTV* gross tumor volume, *AGMs* adrenal gland metastases, *SBRT* stereotactic body radiation therapy

Meanwhile, the median PFS was 4.5 months (95%CI 0.0-9.4 months). The PFS rates at 0.5-, 1-year, and 2- year were 47.4%, 37.9%, and 16.3%, respectively (Fig. 1b).

At the last follow-up, 18 patients (78.3%) died while 5 were alive. One patient died of cirrhosis, whereas 16 patients died of distant metastasis. Hence, local failure and radiation-induced toxicity did not contribute to the death. The median OS was 18.6 months (95%CI 4.7–32.5 months). The 0.5-, 1-year and 2-year OS rates was 97.0%, 88.8%, and 87.0% respectively (Fig. 1c). Regarding the univariate analysis, GTV ≥ 34.5 ml (*p* = 0.011), additional organ metastases after SBRT (*p* = 0.006), and no application of the synchrony respiratory motion tracking method (*p* = 0.038) were strongly associated with poor OS rates. In the meantime, the involvement of targeted therapy (*p* = 0.097) or systemic therapy (*p* = 0.078) may be associated with better OS, but the association was not notable (Table 4). After multivariate analysis, favorable predictors correlated with OS were GTV < 34.5 ml (*p* = 0.043), systemic therapy (*p* = 0.017), and no additional organ metastasis after SBRT (*p* = 0.009) (Table 5).

**Table 4:** Univariate analysis for OS rate

	0.5-year OS rate (%)	1-year OS rate (%)	2-year OS rate (%)	*P* value
Concurrence of metastasis in other sites				
▪ Yes	92.3	67.1	38.4	0.660
▪ No	100.0	66.7	44.4	
GTV (ml)				
▪ < 34.5	100	76.9	53.8	0.011
▪ ≥ 34.5	88.9	50.8	16.9	
Local control				
▪ Yes	94.4	70.8	45.1	0.121
▪ No	100.0	50.0	25.0	
Additional organ metastasis after SBRT				
▪ Yes	94.4	59.0	29.5	0.006
▪ No	100	100	100	
Tracking method				
▪ X-sight spine	95	73.9	45.5	0.038
▪ Synchrony respiratory motion	100.0	50.0	0	
Age (years)				
▪ < 60	93.8	67.0	44.6	0.263
▪ ≥ 60	100	66.7	33.3	
Targeted therapy				
▪ Yes	100	100	60	0.097
▪ No	93.8	53.6	33.5	
Systemic treatment				
▪ Yes	100	100	66.7	0.078
▪ No	93.3	50.3	28.7	

*OS* overall survival, *GTV* gross tumor volume

**Table 5:** Multivariate analysis for OS rate

Variables	Overall survival		
	HR	95% CI	*P* value
GTV (ml)			
< 34.5 vs. ≥ 34.5	0.317	0.104-0.962	0.043
Additional organ metastasis after SBRT			
Yes vs. None	25.195	2.224-285.393	0.009
Systemic treatment			
None vs. Yes	6.453	1.396-29.819	0.017

### Treatment toxicity

The treatment was well tolerated with only 1 patient reporting grade 3 hepatic injury. No grade 4 or higher acute toxicity was observed. Gastrointestinal side effects including nausea, poor appetite, abdominal pain, vomiting, hematochezia, and gastric distension were the most common early toxicities during the follow-up, which resolved spontaneously after SBRT. One patient reported grade 3 late hepatic injury more likely due to targeted drugs, which was improved after hepatoprotective treatment (Table 6).

**Table 6:** Adverse effects reported/observed

AE	Grade 1-2	Grade3 or more	Total N (%)
Nausea	2 (8.7%)	0 (-)	2 (8.7%)
Vomiting	1 (4.3%)	0 (-)	1 (4.3%)
Poor appetite	3 (13.0%)	0 (-)	3 (13.0%)
Diarrhea	0 (-)	0 (-)	0 (-)
Abdominal pain	1 (4.3%)	0 (-)	1 (4.3%)
Hematochezia	1 (4.3%)	0 (-)	1 (4.3%)
Hepatic injury	0 (-)	1 (4.3%)	1 (4.3%)
Fatigue	5 (21.7%)	0 (-)	5 (21.7%)
Gastric distension	1 (4.3%)	0 (-)	1 (4.3%)

*AE* adverse effects

## Discussion

In this retrospective study, we reported the results of SBRT in the treatment of AGMs from liver cancer. Despite the fact that the majority of patients died of distant metastasis, SBRT could possibly provide survival benefits with high LC rates and low toxicity rates, particularly for the small lesions with GTV less than 34.5 ml. No severe adverse events (grade 4 or more) were reported. Despite the limited number of patients enrolled, this was the first two-center study to report CyberKnife in the treatment of AGMs from liver cancer.

The current first-line recommendations for metastatic liver cancer were multiple kinase inhibitors (sorafenib) and systemic anti-programmed cell death protein-1 inhibitors (nivolumab and pembrolizumab) for hepatocellular carcinoma [5, 20, 21]. The ABC-02 trial reported gemcitabine in combination with cisplatin was currently regarded as the best choice for systemic treatment in palliative care for intrahepatic cholangiocarcinoma [22]. Improvements in cancer diagnostic techniques and treatments have resulted in the emergence of a large number of advanced cancer patients, but in the good physical condition and claiming to reduce the tumor burden. Therefore, the treatment strategy relies on multidisciplinary medical practitioners to individualize the patient’s treatment to control systemic diseases. Compared to the common metastatic sites (e.g. bone, lung), adrenal metastases present with relatively few symptoms and few effective options available. Furthermore, an increasing number of liver cancer patients with AGMs were treated with definitive local therapies in a variety of ways.

Previous studies have shown that invasive and micro-invasive treatment of AGMs were associated with better outcomes, with survival ranging from 2 to 21.4 months and 9.3 to 24.9 months respectively after adrenalectomy and thermal ablation [23–26]. However, serious complications after surgery such as pancreatic fistula, adrenal insufficiency, bleeding, and complications due to the technique may occur [3]. In a study of 22 patients with single-sided AGMs who underwent percutaneous ultrasound (US)-guided radiofrequency ablation (RFA), Huang et al. [7] found that local failure rates were 15.8%, 26.3%, and 26.3% at 3, 6, and 12 months respectively after the RFA procedures, with additional OS rates of 79.7%, 52.6%, and 32.9% at 6, 12, and 24 months, respectively. One patient, however, experienced a severe major complication (SIR C) known as myocardial transient ischemia. Lyu et al. [27] analyzed 27 AGMs in hepatocellular carcinoma patients treated with CT-guided thermal ablation. The median follow-up was 19.3 months, and 40.7% of patients exhibited adrenal tumor progression after ablation. The median PFS and OS for the 27 patients were 6.9 months and 16.8 months, respectively. The OS rates at 6-, 12- and 24-month were 88.9%, 66.7%, and 33.3% respectively. In contrast, SBRT was a reliable option for patients who refused or were not suitable for adrenalectomy or micro-invasive treatment. The findings from our study with 24 AGMs from 23 liver cancer patients revealed that the mean LC, median PFS, and OS were 55.8 months, 4.5 months, and 18.6 months, respectively. The 1-year LC, PFS, and OS rates were 77.8%, 37.9%, and 88.8%, respectively. What’s more, patients with small lesions with GTV less than 34.5 ml were associated with better LC and OS. The outcomes were comparable to, if not better than, those of surgery or micro-invasive treatment. Importantly, the toxicities were tolerable, with no cases of grade 4 or higher toxicity reported.

Compared to the invasive and micro-invasive treatments described above, radiotherapy inactivated tumor tissue more gently without a surge of catecholamines, treatment-related hypertension, and adrenal dysfunction [28]. A growing number of studies have focused on radiotherapy in the treatment of AGMs from hepatocellular carcinoma, with conventional radiotherapy being the most used [8–11]. Yuan et al. investigated 81 patients with AGMs from hepatocellular carcinoma, 18 of whom received helical TomoTherapy while 63 patients received conventional radiotherapy. The 2-year OS rate was 35.0% with a median survival time of 15 months. One (1.2%) patient reported grade 3 leucopenia, while 7 (8.6%) patients reported grade 3 thrombocytopenia [11]. In our study, a higher 2-year OS rate with a lower toxicity rate than those corresponding results reported in Yuan’s study. This is probably because SBRT has superior dose distribution compared to conventional radiotherapy, meanwhile, highly conformal and ablative radiation doses could be delivered by SBRT with a low incidence of toxicity. Notably, SBRT has emerged as an alternative to conventional radiotherapy in the management of pain control, which was similar to the conclusions reached in our study [29]. Our study has demonstrated the efficacy and safety of SBRT as an alternative treatment for AGMs.

According to the previous studies on SBRT for AGMs [30–36], the 1-year LC rate ranged from 73 to 97%. It has been shown that an elevated dose of BED_10_ tends to be associated with better local tumor control. One study modeling the probability of LC about SBRT for AGM indicated that the 1-year LC rate was 95% when BED_10_ was equivalent to 116.4 Gy [37]. In addition, a meta-analysis of 39 studies revealed that when BED_10_ were 60, 80, and 100 Gy, the corresponding 1-year LC rates were 70.5%, 84.8%, and 92.9%, respectively; the corresponding 2-year LC rates were 47.8%, 70.1%, and 85.6%, respectively [38]. The median BED_10_ in our study was 72 Gy (53.7-100.8 Gy) with both 1-and 2- year LC rates of 77.8%, which was similar to the findings from other published studies. However, patients with varying BED10 had comparable LC in our study. This is probably influenced by the limited number of patients enrolled as well as the relatively low overall BED_10_ and the large volume of lesions. Different from our study, Ehret et al. analyzed 55 patients with AGMs from different primary tumors, with a median BED_10_ of 80.4 Gy. 1-year and 2-year LC rates were 92.9% and 67.8%, respectively [39], the favorable results might be attributed to the variable primary tumors, relatively small GTV, and high median BED_10_. Voglhuber et al. [40] analyzed 31 patients with 34 AGMs and concluded that PTV volume (PTV < 80 ml, *p* = 0.033) was an indicator of LC, whereas GTV was an indicator of LC in our study.

SBRT for AGMs had mild and low frequent side effects owing to the highly accurate radiation delivery. 1.8% of patients reported grade 3 or higher toxicities while 0.2% of patients reported grade 4 toxicities according to the meta-analysis by Chen et al. [38]. There was only one patient who reported grade 5 toxicity after receiving nivolumab with SBRT [30, 32, 35, 36, 41–43], while in our study, only one patient recorded a grade 3 adverse reaction. Grade 1–2 toxicities including fatigue, nausea, poor appetite, abdominal pain, vomiting, hematochezia, and gastric distension were resolved spontaneously.

Our study had several limitations. Firstly, it was a retrospective study. Secondly, the limited number of patients enrolled could not be able to detect the rare event. Thirdly, the baseline of patients was heterogeneous, such as the variety of lesions by pathological histology. Additionally, the treatment of primary tumors was not thoroughly reviewed and future relevant studies are warranted.

## Conclusion

SBRT is a safe and effective treatment for patients with AGMs from liver cancer, it could provide a high local control rate and mild treatment-related side effects. And patients with small metastatic lesions (less than 34.5 ml) may benefit most at LC and OS. Distant metastases still occurred after SBRT, implying the importance of systemic treatment in high-risk patients.

## Data Availability

The datasets supporting the conclusions of this article are included within the article.

## Abbreviations

SBRT: Stereotactic body radiation therapy
AGMs: Adrenal gland metastases
LC: Local control
PFS: Progression free survival
OS: Overall survival
BED: Biologically effective dose
CTCAE: Common Terminology Criteria for Adverse Events
CR: Complete response
PR: Partial response
SD: Stable disease
PD: Progressive disease
GTV: Gross tumor volume
EHM: Heterochronic extrahepatic metastases
QoL: Quality of life
PDL1: Programmed death-ligand 1
VEGF: Vascular endothelial growth factor
TACE: Transcatheter arterial chemembolization
PEI: Percutaneous ethanol injection
RFA: Radiofrequency ablation
PET/CT: Positron emission tomography /computed tomography
CT: Enhanced computed tomography
MRI: Enhanced magnetic resonance imaging
LR: Left-right
CC: Cranial-caudal
AP: Anterior-posterior
PTV: Planning target volume
RECIST: Response Evaluation Criteria in Solid Tumors
